# Prevalence, Risk Factors, and Antimicrobial Resistance Profile of Respiratory Pathogens Isolated From Suckling Beef Calves to Reprocessing at the Feedlot: A Longitudinal Study

**DOI:** 10.3389/fvets.2021.764701

**Published:** 2021-11-02

**Authors:** Diego Nobrega, Sara Andres-Lasheras, Rahat Zaheer, Tim McAllister, Elizabeth Homerosky, R. Michele Anholt, Craig Dorin

**Affiliations:** ^1^Department of Population Medicine, Ontario Veterinary College, University of Guelph, Guelph, ON, Canada; ^2^Lethbridge Research and Development Centre, Agriculture and Agri-Food Canada, Lethbridge, AB, Canada; ^3^Veterinary Agri-Health Services, Rocky View County, AB, Canada; ^4^One Health at UCalgary, University of Calgary, Calgary, AB, Canada

**Keywords:** antimicrobial resistance, beef calves, bovine respiratory disease, *Mycoplasma bovis*, *Pasteurella multocida*

## Abstract

Here, we investigated the prevalence and risk factors for the presence of *Histophilus somni, Mannheimia haemolytica, Mycoplasma bovis*, and *Pasteurella multocida* in the respiratory tract of calves from the spring processing to the reprocessing at feedlots. Additionally, we characterized, phenotypically and genotypically, the antimicrobial resistance (AMR) profile of the four species. Calves from 22 cow–calf operations were enrolled in the study (*n* = 30 calves per operation) and sampled by deep nasopharyngeal swabs at three time points: spring processing, weaning, or induction into feedlots, and at reprocessing at the feedlot. Isolates were tested for susceptibility using the minimum inhibitory concentration (MIC) test against commonly administered antimicrobials. Additionally, a subset of isolates underwent whole-genome sequencing to infer presence of AMR genes and resistance determinants. Among studied pathogens, *P. multocida* was the most prevalent species, regardless of time point, followed by *M. haemolytica, M. bovis*, and *H. somni*. For *M. bovis*, a sharp increase in prevalence was detected at the reprocessing sampling, whereas for *P. multocida*, an increase in prevalence was observed at the weaning/induction sampling. Comingling and co-location of feedlots were not associated with prevalence of any respiratory pathogen. In terms of AMR, resistance against macrolides was prevalent in *M. bovis*, with most isolates resistant against tildipirosin, tilmicosin, and tylosin. In general, there was limited evidence to support an increase in resistance rates of respiratory bacteria from the spring processing to reprocessing at feedlots, with the exception of florfenicol resistance in *M. bovis*, which increased at reprocessing. Metaphylactic administration of tetracyclines at feedlot induction was not associated with the MIC of tetracyclines in any respiratory bacteria. Conversely, there were clear associations between the parenteral use of macrolides as metaphylaxis at the feedlot induction, and increased MIC against macrolides in *P. multocida, M. haemolytica*, and *H. somni*. Overall, the AMR phenotypes were corroborated by presence of AMR genes. We hypothesize that the administration of macrolides such as tulathromycin at feedlot induction contributes to historical changes in macrolides MIC data of respiratory bacteria of beef cattle.

## Introduction

Antimicrobial resistance (AMR) has emerged as one of the most important threats facing public health globally. By 2050, it is estimated that AMR will claim 10 million human lives per year (1). The rapid dissemination of AMR is aggravated by indiscriminate use of antimicrobials in humans and animals. It is increasingly recognized that the administration of antimicrobials in food-producing animals can contribute to the emergence and spread of antimicrobial resistant strains in animals as well as in humans (2). Accordingly, use of antimicrobials in livestock is under increasing scrutiny.

Bovine respiratory disease (BRD) is one of the leading causes of morbidity and mortality for North American beef cattle (3), and a frequent reason for the use of antimicrobials at feedlots (4). A number of factors can predispose to BRD, including host (age, genetics, and co-infections), agent (causative pathogen), and environmental factors such as transportation of animals, comingling, and extreme weather (5). Among causative agents, *Histophilus somni, Mannheimia haemolytica, Mycoplasma bovis*, and *Pasteurella multocida* are prevalent in clinical BRD (6). BRD is often polymicrobial, with complex interactions between pathogens and the host immune system. The complex nature of BRD infections challenges the accurate identification of cases (7), which when identified are commonly treated with antimicrobials.

Cow–calf operations in Western Canada are mostly extensive and characterized by large pastures in which animals are housed. Antimicrobials commonly administered to treat respiratory disease in Canadian cow–calf cattle include phenicols, tetracyclines, and macrolides (8); the latter are classified as critically important antimicrobials of the highest priority to human health according to the World Health Organization (WHO) (9). Regional increases in resistance rates of BRD pathogens against specific antimicrobial classes have been reported in North America and France (10, 11), which may have been fueled by the administration of antimicrobials and spread of resistant clones (12).

The use of antimicrobials has been implicated as a cause of decreased susceptibility in BRD bacteria from cattle (13). In Western Canadian cow–calf operations, antimicrobials are commonly administered for treatment of lameness in cow and bulls, and for respiratory disease and diarrhea in calves (14). It remains unknown whether and to what extent early-life exposure to antimicrobials can impact pathogen carriage and AMR at the feedlot. At feedlots, antimicrobials are frequently administered to calves as metaphylaxis; e.g., the antimicrobial treatment of a group of animals to prevent or control infectious diseases in high-risk animals at feedlot entry. Metaphylaxis is a highly effective practice to reduce morbidity and mortality of feedlot cattle (15). Arguably, limitations of BRD diagnostics and the ease in which antimicrobials can be administered to calves at feedlots largely contribute to the widespread adoption of metaphylaxis. In terms of administration, many antimicrobial formulations can be used for mass medication either in feed or in drinking water. Alternatively, parenteral (injectable) antimicrobials are also available and used routinely in Western Canadian operations as metaphylaxis (8). Altogether, routine metaphylaxis has led to substantial antimicrobial use as healthy, sometimes low-risk animals will also be treated. Given the increasing recognition of an AMR One Health framework, it is important to increase our understanding of potential impacts of metaphylaxis in AMR of BRD pathogens, which includes the study of potential effects of different antimicrobial classes toward AMR. Such assessment can later incorporate informed discussions on the risks and benefits of metaphylaxis in beef cattle, followed by the establishment of best practices related to the practice.

Our overarching goals were to identify factors associated with prevalence of respiratory pathogens, and to infer potential effects of antimicrobials on AMR in BRD-associated bacteria isolated from suckling beef calves. Specific objectives of this longitudinal study were to (1) estimate prevalence of respiratory pathogens in beef calves from branding through to reprocessing at the feedlot; (2) study potential risk factors for increased carriage of respiratory pathogens; and (3) investigate the AMR profile of respiratory pathogens at different time points, including the study of factors associated with resistance such as the metaphylaxic administration of antimicrobials.

## Materials and Methods

### Ethics Statement

This study followed strict recommendations of the Canadian Council of Animal Care. The research protocol was reviewed and approved by the Lethbridge Research and Development Center's Animal Care Committee (Protocol Review #1639).

### Cow–Calf Operations and Feedlots

Producers were recruited using a client database from a beef cattle veterinary practice in the province of Alberta. Cow–calf operations were eligible for enrolment based on the following criteria: (i) physically located in the province of Alberta; (ii) a minimum herd size of 30 cows; (iii) expected retained ownership of calves after feedlot induction; and (iv) agreement to provide detailed information about the health and antimicrobial use in enrolled calves. Producers were informed that their participation would be anonymous, and they would be financially compensated for the use of their cattle.

Twenty-two cow–calf operations that met the eligibility criteria agreed to participate. Cow–calf operations ranged from 200 to 3,200 cows and were a mixture of pure-bred and mixed-breed commercial operations. From each operation, 30 calves were randomly selected during spring processing (branding) from April to June 2017. Calves were selected using a systematic random approach, where every *k*th calf pulled for processing at a cow–calf operation was selected. *k* was defined as the nearest integer of the quotient of *N* over 30, where *N* stands for the number of calves to be processed in the operation. Calves selected were clinically healthy and had not been exposed to antimicrobials. Assuming a low within-herd prevalence of respiratory pathogens in pre-weaned calves (16), an intraclass correlation coefficient of 0.25, and an error rate of 5%, sampling 30 animals per operation allowed us to estimate a prevalence of 7.65% of any BRD-associated pathogen with 90% power at any given time point. The study had 76% power to detect an odds ratio of 2 associated with an increased risk of AMR in respiratory pathogens isolated from calves that received antibiotics at feedlot induction. This was based on the assumption that 6.3% of feedlot cattle would be BDR-positive (17), half of feedlots employed metaphylaxis (18), a baseline prevalence of any given resistance of 20% in respiratory pathogens, and a standard deviation of operation-level random effects of 2.

Management protocols were determined by each producer. The most common procedures at spring processing included vaccination [modified-live BVD type 1 (Bovine Viral Diarrhea Virus) and 2/IBR/PI3/BRSV (Infectious Bovine Rhinotracheitis Virus, Parainfluenza Virus 3, Bovine Respiratory Syncytial Virus) vaccine, seven- or eight-way clostridial, *H. somni*, and *M. haemolytica* vaccines] and castration of males. The spring processing branding protocol of some operations included dehorning, growth implants, and/or administration of meloxicam. Calves were fed on the dam's milk and had access to forage. Calves were weaned in the fall and moved to a feedlot. All calves underwent “hard” weaning where they were abruptly separated from dams. Calves from six operations were moved to a distant feedlot (<1–3 h of driving). In the remaining 16 operations, calves were placed in a feedlot co-located with the cow–calf operation. A typical processing protocol for fall-placed, high-risk calves included a modified-live BVD type 1 and 2/IBR/PI3/BRSV vaccine, seven- or eight-way clostridial bacterin, *H. somni* bacterin, *M. haemolytica* bacterin, endectocide, and an anabolic implant. At feedlots, adoption of metaphylaxis varied by producer, and this information was recorded. Animals were housed according to sex in large outdoor dirt-floor pens with porosity fencing. Calves were fed rations once or twice daily, which were formulated to meet standard nutritional requirements of backgrounding or finishing feedlot cattle.

### Sampling Protocol

Samples were collected at three time points: (i) spring processing (BRANDING) when calves were from 2 to 8 weeks old; (ii) at weaning or feedlot induction (WEANING/INDUCTION), with animals aged from 5 to 8 months old; and (iii) at reprocessing at the feedlot (REPROCESSING) when calves were from 9 months to 1 year of age. From each animal, deep nasopharyngeal swabs (DNPS) were collected as described (Supplementary Methods). Following sample collection, guarded culture swabs (CP Group, Newmarket, ON) were placed in Amies culture media (ThermoFisher Scientific, Mississauga, ON) and transported to the laboratory.

At WEANING/INDUCTION, timing of sampling was not consistent between herds. As rapid changes in nasopharyngeal microbiota following arrival at the feedlot are expected (19), it was important to distinguish samples collected at weaning at the cow–calf environment from samples collected post-weaning after arrival at the feedlot. Producers were asked if calves were comingled with calves from other sources at the feedlot and, if so, when the DNPS was collected relative to the time of transport and comingling. Calves sampled at weaning, prior to or within 24 h of feedlot arrival were considered to be “not comingled” at WEANING/INDUCTION. Additionally, calves from producers that did not introduce animals from other sources at their own feedlots were also considered to be “not comingled” at WEANING/INDUCTION. All other calves were considered “comingled.” At REPROCESSING, all animals were considered “comingled” except in operations where producers fed their own animals without introducing animals from other sources.

Number of days on feed (DOF) at feedlots was obtained for each calf. Additionally, all antimicrobial treatments were recorded, from birth up to REPROCESSING, including the reason for treatment and the type of antimicrobial administered. Pasture (pre-weaning) treatments, metaphylactic antimicrobial use at feedlot induction, and therapeutic antimicrobial use during feeding were recorded. Antimicrobial use data at feedlots were recovered from electronic management systems. Where producer records clearly stated “no antimicrobials,” AMU exposure was considered as “none.” Where farm records were either not provided or unclear regarding exposure to antimicrobials, the associated AMU history of an animal was classified as “unknown.” Decisions involving antimicrobial use were made by producers with support from veterinarians.

### Bacteriology

After arrival at the laboratory, each swab was individually immersed in 1 ml of brain–heart infusion (BHI; BD) containing 20% glycerol and vortexed for 1 min. A 50-μl aliquot was plated onto tryptic soy agar with 5% sheep blood (BAP; Dalynn Biologicals, Calgary, AB, Canada) for the isolation of *H. somni*. A second aliquot (100 μl) was plated on blood agar supplemented with 15 μg/ml bacitracin (BAC; Dalynn) for the isolation of *M. haemolytica* and *P. multocida*. BAC plates were incubated in an aerobic atmosphere at 37°C for 24 h, and examined for the presence of suspected *P. multocida* and *M. haemolytica* colonies, whereas BAP plates were incubated for 2 days in a 5% CO_2_ atmosphere before examination of *H. somni* colonies. When *M. haemolytica, P. multocida*, or *H. somni* suspected colonies were observed in primary cultures (20), three colonies were first sub-cultured onto separate BAP plates, from which one plate per sample/species was randomly selected for further characterization. Bacteria were stored at −80°C in BHI supplemented with 20% glycerol for further analysis.

For the isolation of *M. bovis*, a 150-μl aliquot of the initial BHI-glycerol suspension was inoculated into 1.5 ml of PPLO broth (Dalynn Biologicals) (21) containing 500 μg/ml of ampicillin (Millipore Sigma, Oakville ON). The mixture was filter sterilized and incubated at 37°C in a 5% CO_2_ atmosphere. After 5 days of incubation, 100 μl of enrichment was plated onto PPLO Agar + ampicillin plates. Plates were incubated for an additional 5 days under the same conditions and primary agar cultures were observed under a stereoscopic microscope. When bacterial growth was observed, an isolated colony was randomly selected and transferred to 1.5 ml PPLO + ampicillin broth. *M. bovis* positive PPLO broth cultures were stored at −80°C in PPLO supplemented with 20% glycerol and 0.5% pyruvate for further analysis.

Species confirmation was carried out for all isolates using PCR following protocols that were internally validated at the Lethbridge Research and Development Center (Table 1). Colonies were suspended in 100 μl of TE buffer (10 mM Tris, 1 mM EDTA, pH 8) and heated for 5 min at 95°C for DNA extraction. The lysate was vortexed and centrifuged, and 2 μl of the supernatant was used as DNA template in separate reactions for each species (Table 1). In all reactions, the HotStartTaq Plus Master Mix kit (Qiagen, Toronto, ON) was used.

**Table 1:** Oligonucleotide primers, PCR protocols, and amplicon sizes for each cPCR assay.

**Bacteria, amplified gene**	**Primer sequences (5^′^-3^′^)^a^**	**Cycling conditions**	**Amplicon size (bp)**	**Primer reference**
*Mannheimia haemolytica, lkt*	F: GTCCCTGTGTTTTCATTATAAG	95°C, 5 min; (94°C, 30 s; 58°C, 45 s; 72°C, 60 s) ×35 cycles; 72°C, 10 min	385	(22)
	R: CACTCGATAATTATTCTAAATTAG			
*Pasteurella multocida*, 23S rRNA	F: GGCTGGGAAGCCAAATCAAAG	95°C, 5 min; (94°C, 30 s; 58°C, 45 s; 72°C, 60 s) ×35 cycles; 72°C, 10 min	1,432	(23)
	R: CGAGGGACTACAATTACTGTAA			
*Histophilus somni*, 16S rRNA	F: GAAGGCGATTAGTTTAAGAG	95°C, 5 min; (94°C, 30 s; 55°C, 45 s; 72°C, 60 s) ×35 cycles; 72°C, 10 min	400	(24)
	R: TTCGGGCACCAAGTRTTCA			
*Mycoplasma bovis, uvrC*	F: TTACGCAAGAGAATGCTTCA	95°C, 5 min; (94°C, 30 s; 56°C, 45s; 72°C, 60 s) ×35 cycles, 72°C, 10 min95°C, 5 min; (94°C, 30 s; 56°C, 45s; 72°C, 60 s) ×35 cycles, 72°C, 10 min	171	(25)
	R: TCATCCAAAAGCAAAATGTTAAA			
*Mycoplasma bovis*, 16S	F: GGGAGCAAACAGGATTAGATACCCT		269	
	R: TGCACCATCTGTCACTCTGTTAACCT			

a *F, forward primer; R, reverse primer*.

### Antimicrobial Susceptibility Testing

Antimicrobial susceptibility testing (AST) was carried out using broth microdilution. For *M. haemolytica* and *H. somni*, all isolates were tested, whereas 150 *P. multocida* isolates were randomly selected for AST. Commercial antimicrobial panels (Thermo Scientific, Mississauga, ON) were used, and tests were carried out according to the manufacturer guidelines. In brief, bacterial inocula were concentration-adjusted in either saline solution (*P. multocida* and *M. haemolytica*) (26) or Mueller-Hinton and yeast extract broth (*H. somni*). The working solutions were inoculated into 96-microwell commercial plates (50 μl per well for *P. multocida* and *M. haemolytica*; 100 μl per well for *H. somni*) that contained a series of two-fold dilutions of antimicrobials of interest (SensititreTM bovine/porcine plate format BOPO6F for *H. somni* and *M. haemolytica*; SensititreTM bovine/porcine plate format BOPO7F for *P. multocida*). Following incubation (35°C for 20 h for *M. haemolytica* and *P. multocida*; 35°C for 24 h in a 5% CO_2_ incubator for *H. somni*), plates were visually examined for presence of bacterial growth. The minimum inhibitory concentration (MIC) was defined on a pathogen and antimicrobial basis according to the Clinical and Laboratory Standards Institute guidelines (27). Additionally, isolates were classified as susceptible, intermediate, or resistant against antimicrobials for which cattle-specific breakpoints have been defined (26). For the purpose of analysis, intermediate and resistant isolates were classified as resistant (or non-susceptible). Antimicrobials, concentrations tested, and breakpoints adopted are listed as a Supplementary Material (Supplementary Table 1).

For *M. bovis*, antimicrobial susceptibility testing was performed as suggested elsewhere (28, 29). Customized antimicrobial plates (30) (Trek Diagnostics, Oakwood, GA, USA) were used to test those antimicrobials that are most relevant for the treatment of *M. bovis* infections in feedlot cattle in western Canada (Supplementary Table 2). The AST custom plate contained 50 μl of a PPLO (21) suspension with antimicrobials in each well. *M. bovis* colonies were grown in PPLO broth with pyruvate for 72 h, and 500 μl of this culture was transferred to 500 μl of fresh PPLO and incubated for another 48 h. A 50-μl aliquot from this final solution was inoculated into each well of the AST plate yielding a concentration of 1 × 10^3^-1 × 10^5^ CFU/ml per well. Plates were incubated for 48 h at 37°C and 5% CO_2_. Results were recorded after 48 h and used to estimate the MIC (31). *M. bovis* ATCC 25523 was used as an internal quality control strain in all assays. Breakpoints were used to classify isolates as susceptible, intermediate, or resistant, as described elsewhere (30) (Supplementary Table 2).

### Sequencing

Eighty-three isolates from BRD *Pasteurellaceae* species [*M. haemolytica* (*n* = 25), *P. multocida* (*n* = 29), and *H. somni* (*n* = 29)] were selected for sequencing. The selection protocol was based on inclusion of isolates of varying MIC levels (low, intermediate, and high) against selected antimicrobials (macrolides, fluoroquinolones, tetracyclines, and phenicols), from different operations and sampling points in order to ensure that all operations were represented. Isolates were streaked onto BAC plates for *M. haemolytica* and *P. multocida*, and BAP plates for *H. somni*. Plates were incubated overnight at 37°C (BAC), or for 48 h at 37°C in 5% CO_2_ (BAP). A single colony was then sub-cultured onto BAC or BAP plates, and incubated as described above. Bacteria were diluted in TE (10 mM Tris, 1 mM EDTA), pH 8.0 buffer to an OD 600 of ≈2, equivalent to ≈2 × 10^9^ cells/ml. The cell suspension (1 ml) was transferred to a microcentrifuge tube and centrifuged for 2 min at 14,000 *g*. Genomic DNA was extracted using DNeasy Blood and Tissue kit (Qiagen, Montreal, QC, Canada) following manufacturer's instructions. DNA quality and quantity were estimated using a Nanodrop 2000 spectrophotometer and a Qubit Fluorometer with PicoGreen (Thermo Fisher Scientific, Mississauga, ON), respectively. Genomic library construction was performed using the Illumina Nextera XT DNA sample preparation kit (Illumina Inc., San Diego, CA, USA). Libraries were sequenced on an Illumina MiSeq platform using the MiSeq Reagent Kit V3 to generate 2 × 300 base paired-end reads.

Sequencing reads were *de novo* assembled into contigs using SPAdes version 3.13.0 with a multi-sized de Bruijn graph approach (32). Draft genome assemblies were annotated with Prokka (33). ABRicate version 0.8.7 (34) was used to screen contigs against the NCBI Bacterial Antimicrobial Resistance Reference Gene Database (NCBI BioProject ID: PRJNA313047) for presence of AMR genes. The sequencing data of isolates used in this study have been submitted to the NCBI (under BioProject ID: PRJNA720670).

### Statistical Analysis

All analyses were carried out in R (35) using the following packages: *brms, lme4, mice*, and *runjags* (36–39).

#### MIC50 and MIC90

Minimum inhibitory concentration results (in μg/ml) were summarized for each pathogen, sampling point, and antimicrobial tested using distribution tables. Likewise, the 50th and 90th MIC percentiles, defined as the MIC capable of inhibiting the growth of 50 and 90% of isolates (MIC_50_ and MIC_90_, respectively), were estimated for each species individually.

#### Prevalence of Respiratory Pathogens and Associated Risk Factors

Prevalence of respiratory pathogens (*P. multocida, M. haemolytica, H. somni*, and *M. bovis*) was estimated at each sampling point (BRANDING, WEANING/INDUCTION, and REPROCESSING). Prior to model estimation, preliminary assessments were carried out to check for missing values. In 21 out of the 22 operations enrolled, at least 1 animal was lost to follow-up either at WEANING/INDUCTION or REPROCESSING (Supplementary Table 3). All calves from five cow–calf operations were lost to follow-up at REPROCESSING. Operation-conditional comparisons of prevalence of respiratory pathogens at WEANING/INDUCTION (the second sampling point) indicated no difference between calves from operations with at least one sample collected at REPROCESSING and calves from the five operations with no REPROCESSING data, meaning that the probability of missing values was most likely independent from the observed values (results not shown). Regardless, data from the five operations were excluded from prevalence estimates at the last sampling point (no imputation was attempted). For the remaining data, we used a multivariate imputation method based on chained equations for handling missing values. Imputation models were used to generate a set of 20 datasets with complete outcome information after 100 iterations each. Imputation was based on the “*2l.bin*” method from the *mice* package in R (38). Imputation models contained operation-level random effects, sex, and results from previous sampling(s) introduced as fixed effects. All Markov chains were visually inspected for convergence, where absence of trends for any chain was deemed adequate. If trends were detected for any inputted parameter, the number of iterations was increased until convergence was achieved. As samples were mostly collected in batches, DOF of missing samples were deemed as the most frequently observed value for samples that were collected at the same feedlot at that sampling. Likewise, a similar approach was used to infer the comingling status of missing samples. Thereafter, generalized linear mixed models with logit link and operation-specific random effects at the intercept level were fit in a Bayesian framework to estimate prevalence of respiratory pathogens, and to study associations between prevalence and potential risk factors [presence of feedlot onsite, sex, comingling status at weaning and results from previous sampling point(s)]. Binary indicators for potential risk factors were generated and introduced as predictors in multivariable models. Risk factor effects were assumed to be common to all levels of remaining model terms (no two- or three-way terms were considered). A minimum of five observations per factor was required for risk assessment; for instance, effects of previous sampling points at WEANING/INDUCTION were not assessed in absence of at least five calves harboring the bacteria at BRANDING. To account for potential deviations from original sampling protocols, DOF centered at 0 and 139 days (median DOF of REPROCESSING samples) were forced in WEANING/INDUCTION and REPROCESSING models, respectively. The relationship between the log odds of the presence of a respiratory pathogen and DOF was assumed to be linear. As DNPS is not a perfect test, latent class models, containing sensitivity estimates based on findings from previous research (40) (Supplementary Table 4), were used to account for potential misclassification of DNPS to detect respiratory pathogens in cattle. For *P. multocida* and *H. somni*, DNPS sensitivity was assumed to be identical to that of *M. haemolytica*. To allow for some degree of uncertainty in sensitivity parameters, beta distributions were truncated at ±5 percentual points from distribution modes. Specificity of DNPS was deemed to be 100%, as results were PCR-confirmed. Non-informative priors were used for all other parameters. Overlapping of credible intervals (95% CI) were used for statistical inference. Predictors not associated with presence of a respiratory pathogen were excluded and simpler models were attempted. A full Bayesian statistical inference framework with Markov chains based on Hamiltonian Monte Carlo sampling was used for model estimation. This scheme generates proposal distributions that are pulled toward the posterior distribution mode instead of being symmetrical around the current position. Two parallel chains per dataset were used with a total of 100,000 post-warmup samples. Effective sample sizes (minimum of 1,000 for each parameter), Rhat estimates per dataset, and visual inspection of chains were used to evaluate efficacy. Posterior distribution plots were generated and distribution modes as well as respective 95% CIs were reported as proportions. Analysis were done in R with use of functions from the *brms* package (36).

A second approach was attempted with the use of models for longitudinal data (three-level models [operation, animal, and sample] with random slopes for time at the second level) and time splines that would allow for the evaluation of non-linear time changes and comingling effects when DOF > 0. In this second approach, DOF was used as a metric of time, where DOF = 0 represented the day when animals transitioned to feeding. However, Markov chains failed to converge. Therefore, results from the first approach were kept for presentation.

#### Antimicrobial Resistance of Respiratory Pathogens

Frequency tables were built to summarize AMR rates for each species and sampling point. Next, two distinct approaches were used to compare resistance rates between sampling points and estimate comingling effects: mixed models and exact logistic regression models. For these analyses, only antimicrobials for which animal-specific breakpoints were available were retained.

Generalized linear mixed effects models were used to estimate effects of comingling and time in the AMR of *P. multocida* at the isolate level. A binary indicator related to presence or absence of resistant *P. multocida* in the sample was considered an outcome. The logit link was used for analysis, and models contained operation-specific random effects. Models were fit for each antimicrobial separately. Comingling effects were analyzed at WEANING/INDUCTION exclusively; only two producers did not comingle their calves at feedlots and it was impossible to distinguish operation-specific effects from comingle effects at REPROCESSING. As calves could not have been comingled prior to BRANDING, models were fit separately to assess time and comingle effects. For time models, results obtained at BRANDING were used as a reference. Models were fit using maximum likelihood, using the adaptive Gauss-Hermite quadrature with 50 quadrature points per scalar (37), providing existence of at least one resistant and one susceptible isolate per strata (sampling point or comingle status), as well as a minimum of five isolates per strata.

For *M. haemolytica, H. somni*, and *M. bovis*, exact logistic regression models were used to compare the prevalence of AMR between sampling points and estimate comingling effects. Exact logistic regression models for clustered data were carried out according to Troxler et al. (41) in R. Pairwise comparisons between sampling points were carried out on a pathogen basis for each antimicrobial in separate analyses. Comingling effects at WEANING/INDUCTION were also tested using exact logistic regression models. Models were attempted providing existence of at least one resistant and one susceptible isolate per species, as well as a minimum of five isolates per strata. Statistical significance was set at the 5% level.

#### Effects of Administration of Antimicrobials in the MIC of Respiratory Pathogens

The goal of this analysis was to compare the MIC of respiratory pathogens sampled from animals treated with antimicrobials to corresponding values from animals not treated. In short, throughout the study, antimicrobials were administered as one of the following: (i) prior to weaning to treat clinical diseases; (ii) metaphylactic injections at weaning or immediately after placement at feedlots; (iii) in feed as BRD metaphylaxis to high-risk groups at feedlot induction; (iv) after feedlot induction, as treatment or prevention of clinical diseases at the feedlot; (v) a second injection to treat clinical diseases at feedlots. Prior to any assessment, records containing unreliable information about antimicrobial exposure status (e.g., where AMU was classified as “unknown”) were excluded. Administration of antimicrobials prior to BRANDING and after WEANING/INDUCTION was uncommon and therefore not analyzed due to limited statistical power.

For the assessment of effects of metaphylactic use of antimicrobials at feedlot induction toward AMR at REPROCESSING, analyses were carried out at the isolate level. A preliminary assessment was carried out at the operation level to compare the distribution of DOF of samples collected at REPROCESSING according to the type of metaphylaxis adopted using general linear models. No differences were detected in DOF of samples collected at REPROCESSING when contrasting operations according to main categories of metaphylaxis (mean DOF of 135, 139, 138, and 135 for calves that were fed chlortetracycline, were not fed chlortetracycline, were treated parenterally with macrolides, and were not treated parenterally with macrolides, respectively; *p* > 0.05). Thereafter, DOF was omitted from further analysis. Isolates obtained at REPROCESSING were retained, and effects of parenteral administration of macrolides or in-feed administration of tetracyclines toward antimicrobial-specific MIC data were assessed using Bayesian models (42). Minor modifications were implemented to adapt the WinBUGS code to JAGS. In brief, log-transformed MIC values (logMIC) were assumed to follow a normal distribution with a common variance. As the true underlying MICs were not observed, logMIC values were considered censored at the maximum and minimum antimicrobial concentrations tested (interval censored). Unlike the original WinBUGS code used for analysis of MIC data (42), the distributions were not truncated in JAGS using the *I* operator. Instead, the *dinterval* function was used to represent censored outcomes, as recommended (43). Models contained frailty terms to account for the variability due to unobserved operation-level effects. Models were fit for tetracyclines and macrolides separately. Fixed-effects included the parenteral administration of macrolides (macrolide models), or the in-feed use of tetracyclines at feedlot induction (tetracycline models). To evaluate effects of other routes of administration that were adopted throughout the study (parenteral administration of tetracyclines or in-feed administration of macrolides), we performed sensitivity analysis. Estimates and conclusions obtained from models with and without observations from selected operations were contrasted. A sensitivity analyses was undertaken due to the absence or low number of operations in each stratum defined according to type of antimicrobial therapy adopted, as this would preclude the proper estimation of operation-level random effects if a second herd-level exposure was added to the models.

Models were attempted when a minimum degree of variability in MIC values was apparent and not analyzed for antimicrobials with the same MIC for all isolates. Models were fit for each pathogen (*M. haemolytica, P. multocida, H. somni*, and *M. bovis*) and antimicrobial [(oxy)tetracycline, tylosin, tulathromycin, tilmicosin, and tildipirosin] combination in a Bayesian framework using a Markov Chain Monte Carlo approach based on Gibbs sampling. Four chains were run in parallel with a total of 500,000 iterations using the *runjags* package (39). Posterior distribution plots were visualized, and 95% CIs were used for statistical inference. Iteration plots were visually inspected for proper mixing of Markov chains. Autocorrelation values and ESS were used as measures of efficacy, where an ESS of 10,000 or higher was deemed as adequate.

## Results

In total, 660 calves were enrolled consisting of 301 heifers and 359 steers. Nearly 40% of calves (*n* = 265) were not sampled at all times (Supplementary Table 3), with the sale of calves as the most common reason for missingness (65% or 173 missing animals), including all calves from five operations, which were sold prior to the third sampling. Additionally, in large operations, study animals were distributed among several pens. In those settings, there were challenges involved in gathering calves for sampling, which accounted for another 87 animals (32% of 265 missing animals) lost to follow-up (Supplementary Figure 1).

Six operations (27%) had a feedlot co-located to the cow–calf operation. At WEANING/INDUCTION, seven producers sampled calves prior to arrival at the feedlot. Nine operations sampled calves after comingling participants with calves from other sources. Following feedlot induction, two producers did not practice comingling, meaning that no animals from other sources were introduced at their feedlots. REPROCESSING samples were collected from 81 to 228 DOF, with an average of 142.6 DOF.

Antimicrobial administration prior to or at BRANDING was limited. A total of four calves from two operations were treated with florfenicol for pneumonia. Additionally, one calf with pneumonia was treated with tilmicosin, and a calf with a navel infection was treated with enrofloxacin. Finally, a calf with footrot was treated with tulathromycin. In total, treatment with antimicrobials prior to or at BRANDING were reported from four operations.

At WEANING/INDUCTION, animals from five operations were categorized as “low risk” and were not treated with antimicrobials. Calves from remaining operations were treated with tetracyclines, macrolides, and a combination of the two classes (Table 2).

**Table 2:** Antimicrobial treatment protocols adopted at WEANING/INDUCTION at each cow–calf operation.

**Operation^a^**	**Feed^b^**		**Parenteral^b^**	
	**Antimicrobial**	**Dosage**	**Antimicrobial**	**Dosage**
1	–^c^	–	–	–
2	Chlortetracycline	1 g/100 lbs^d^	Gamithromycin	6 mg/kg
3	–	–	Tulathromycin	2.5 mg/kg
5	–	–	–	–
7	Chlortetracycline	1 g/100 lbs^d^	Tulathromycin	2.5 mg/kg
9	–	–	–	–
11	–	–	–	–
12	Chlortetracycline	1 g/100 lbs^d^	Tulathromycin	2.5 mg/kg
13	–	–	Tilmicosin	10 mg/kg
15	–	–	Oxytetracycline	20 mg/kg
16	Chlortetracycline	1 g/100 lbs^d^	Tilmicosin^e^	10 mg/kg^e^
17	–	–	–	–
18	Chlortetracycline	1 g/100 lbs^d^		
19	–	–	Tulathromycin	2.5 mg/kg
20	Chlortetracycline	1 g/100 lbs^d^	Oxytetracycline	20 mg/kg
21	Chlortetracycline	1 g/100 lbs^d^	–	–
22	Chlortetracycline	1 g/100 lbs^d^	–	–

a *Five operations with no antimicrobial use data were excluded*.

b *Protocols listed are those adopted in most calves from each operation*.

c *A dash (–) indicates that antimicrobials were not used at WEANING/INDUCTION by that route of administration*.

d *Body weight, daily for 15 days*.

e *Protocol administered to 15 calves. Another 14 calves were treated with tulathromycin (2.5 mg/kg)*.

After induction, calves from seven operations received no antimicrobials. Two operations that induced with macrolides also administered tylosin in feed to prevent or reduce the incidence of liver abscesses (110 mg/hd-day). Fourteen of the 392 (3.6%) calves that remained in the study were treated individually with antimicrobials at least once after induction. From this total, nine calves from four feedlots were treated for BRD. Other reasons for treatment included lameness (*n* = 3), footrot (*n* = 2), atypical interstitial pneumonia (*n* = 1), and infectious keratoconjunctivitis (*n* = 1). The most commonly used antimicrobials were florfenicol (*n* = 8 animals; four operations), ceftiofur (*n* = 4; two operations), and tilmicosin (*n* = 3; one operation).

### Prevalence of Respiratory Pathogens and Associated Risk Factors

**Figure 1:**
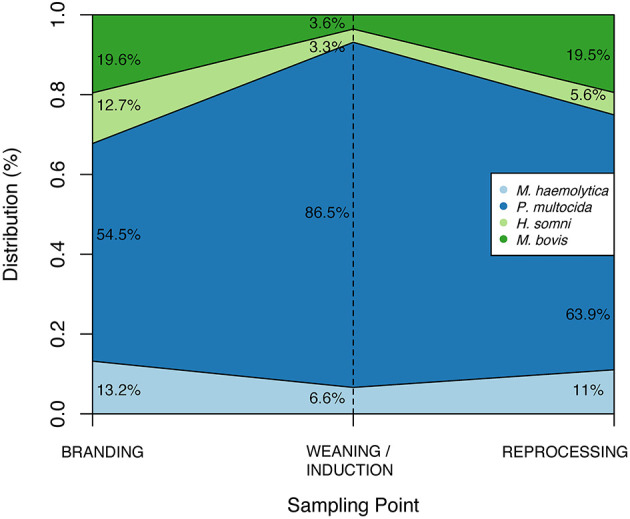
Distribution of respiratory pathogens according to sample point.

Among studied BRD-associated pathogens, *P. multocida* was the most frequently recovered species (*n* = 424), followed by *M. haemolytica* (*n* = 61), *M. bovis* (*n* = 49), and *H. somni* (*n* = 30). From a total of 660 samples collected at BRANDING, 7.4% (*n* = 49) were positive for at least one respiratory pathogen. This percentage increased to 52.3% (310/608) at WEANING/INDUCTION, and 50.3% (197/392) at REPROCESSING at feedlots. Accordingly, prevalence of *P. multocida, M. bovis*, and *M. haemolytica* were significantly higher at REPROCESSING than at BRANDING (Table 3). Additionally, for *M. haemolytica* there was no difference between prevalence at WEANING/INDUCTION, and prevalence at either BRANDING or REPROCESSING. Conversely, in *M. bovis*, there was a significant difference between prevalence at WEANING/INDUCTION and at REPROCESSING, suggesting that animals were infected at feedlots. For *P. multocida*, a sharp increase in prevalence was observed at WEANING/INDUCTION, which persisted at REPROCESSING (Figure 1). *M. bovis* was the second most prevalent respiratory pathogen at feedlots (Figure 1). Comingling, co-location of feedlot, and cow–calf operation, and results from previous sampling point did not have an impact on the prevalence of respiratory pathogens at WEANING/INDUCTION.

**Table 3:** Prevalence per 100 animals of *Mannheimia haemolytica, Pasteurella multocida, Histophilus somni*, and *Mycoplasma bovis* at BRANDING (*n* = 660 samples), WEANING/INDUCTION (*n* = 608 samples), and REPROCESSING (*n* = 392 samples).

**Bacteria**	**BRANDING**		**WEANING/INDUCTION**		**REPROCESSING**	
	**Prevalence**	**95% CI^1^**	**Prevalence**	**95% CI**	**Prevalence**	**95% CI**
*M. haemolytica*	0.87^a^	0.68; 1.47	3.06^a,b^	1.20; 6.19	5.31^b^	2.28; 9.96
*P. multocida*	3.59^a^	1.34; 7.71	40.0^b^	27.9; 49.2	30.74^b^	22.04; 40.36
*H. somni*	0.83	0.67; 1.35	1.53	0.72; 3.52	2.70	0.88; 7.13
*M. bovis*	1.29^a^	0.70; 2.80	1.65^a^	0.71; 4.71	9.37^b^	4.92; 14.99

1 *95% Credible interval (95% CI)*.

a,b *Within row prevalences followed by different letters denote statistically significant differences between sampling points for each species*.

### Antimicrobial Resistance of Respiratory Pathogens

On average, 14 isolates per operation were tested for susceptibility, ranging from 5 to 28 isolates per operation. MIC_50_ and MIC_90_ according to species and sampling point are available as Supplementary Material (Supplementary Tables 5–8). Overall, low levels of AMR were detected in BRD-associated *Pasteurellaceae* species isolates. Considering antimicrobials for which resistance breakpoints have been established, resistance against spectinomycin was observed in 12, 11, and 3% of *M. haemolytica, P. multocida*, and *H. somni* isolates, respectively. In *M. bovis*, resistance against macrolides was prevalent, with the majority of isolates resistant to tildipirosin, tilmicosin, and tylosin. In contrast, resistance to enrofloxacin and ceftiofur was absent or rare, regardless of species (Table 4). Resistance to tetracyclines was observed in all species, ranging from 3.3% of oxytetracycline resistance in *H. somni* to 18.4% in *M. bovis*. Resistance to tetracyclines and spectinomycin appeared to co-exist in *M. haemolytica, P. multocida*, and *H. somni*.

**Table 4:** Antimicrobial resistance rates (%) according to antimicrobial, sampling point (B, BRANDING; I, WEANING/INDUCTION; R, REPROCESSING), and bacteria species.

**Class**	**Antimicrobial**	***Mannheimia haemolytica***			***Histophilus somni***		
		**B**	**I**	**R**	**B**	**I**	**R**
		***n*** **= 1**	***n*** **= 30**	***n*** **= 27**	***n*** **= 0**	***n*** **= 10**	***n*** **= 20**
Aminocyclitol	Spectinomycin	0	20	3.7	–^1^	10	0
Cephalosporin	Ceftiofur	0	0	0	–	0	0
Fluoroquinolone	Danofloxacin	0	3.3	0	–	10	5
	Enrofloxacin	0	0	0	–	10	0
Macrolide	Tilmicosin	0	13.3	14.8	–	10	0
	Tulathromycin	0	0	3.7	–	0	0
Penicillin	Penicillin	0	3.3	14.8	–	0	0
Phenicol	Florfenicol	0	0	0	–	10	0
Tetracycline	Oxytetracycline	0	0	7.4	–	0	5
**Antimicrobial**	**Antimicrobial**	***Pasteurella multocida***			***Mycoplasma bovis***		
		**B**	**I**	**R**	**B**	**I**	**R**
		***n*** **= 24**	***n*** **= 88**	***n*** **= 38**	***n*** **= 2**	***n*** **= 19**	***n*** **= 28**
Aminocyclitol	Spectinomycin	8.3	10.2	15.8	–	–	–
Cephalosporin	Ceftiofur	0	0	0	–	–	–
Fluoroquinolone	Danofloxacin	0	0	0	–	–	–
	Enrofloxacin	0	0	0	0	0	0
Macrolide	Gamithromycin	0	1.1	5.3	100	84.2	100
	Tildipirosin	0	0	5.3	100	94.7	100
	Tilmicosin	4.2	3.4	7.9	100	94.7	100
	Tulathromycin	0	0	5.3	100	31.6	60.7
	Tylosin	–	–	–	100	78.9	85.7
Penicillin	Penicillin	0	0	0	–	–	–
Phenicol	Florfenicol	0	0	0	50	36.8^a^	67.9^b^
Tetracycline	Chlortetracycline	–	–	–	0	15.8	14.3
	Oxytetracycline	–	–	–	0	15.8	21.4
	Tetracycline	8.3	9.1	15.8	–	–	–

1 *A dash (–) denotes either absence of clinical breakpoints for that specific pathogen–antimicrobial combination or absence of tested isolates*.

a,b *Within row percentages followed by different letters denote statistically significant differences between resistance rates for each species (p < 0.05)*.

There was limited evidence to support an increase in resistance rates from BRANDING to REPROCESSING in BRD-associated bacteria isolated from cattle (Table 4). Most pairwise comparisons indicated no significant differences in AMR rates among sampling points. The exception was resistance against florfenicol in *M. bovis*; 68% of isolates at REPROCESSING were resistant against florfenicol vs. 37% of *M. bovis* isolated at WEANING/INDUCTION (*p* = 0.02). Florfenicol-resistant *M. bovis* were isolated from eight feedlots. From this total, only a single feedlot reported one incidence of treatment with florfenicol. Finally, there were no differences in resistance rates at WEANING/INDUCTION among bacteria isolated from comingled and non-comingled calves.

As inferred from the WGS, 2 out of 25 sequenced *M. haemolytica* isolates harbored the *aph(3*′*)-Ia, aph(6)-Id, aph(3*″*)-Ib, sul2*, and *tet(H)* antimicrobial resistance genes (ARGs). Additionally, 9 out of 29 sequenced *P. multocida* had the *aph(3*′*)-Ia, aph(6)-Id, aph(3*″*)-Ib, sul2, aadA31*, and *tet(H)* genes, with two isolates also carrying a A2058G mutation in the 23S rRNA gene. We did not detect any ARG in *H. somni* sequenced isolates. Overall, the AMR phenotypes were corroborated by presence of ARGs (Supplementary Table 9). The *tet*(*H*) gene was present in all tetracycline-resistant *M. haemolytica* and *P. multocida*. *P. multocida* and *M. haemolytica* isolates harboring the neomycin and kanamycin resistance gene *aph(3*′*)-Ia* had neomycin MIC values >32 μg/ml. *aadA*31 was associated with spectinomycin resistance in *P. multocida* (Supplementary Table 9). The streptomycin resistance ARGs *aph(3*″*)-Ib* and *aph(6)-Id* were detected in all *M. haemolytica* and *P. multocida* strains harboring the neomycin resistance determinant *aph(3*′*)-Ia*. Unfortunately, streptomycin resistance was not assessed for any species. In all isolates, the MIC of sulphadimethoxime was ≥256 μl/ml. Yet, *sul2* was detected in 9 *P. multocida* and 2 *M. haemolytica* isolates (31.0 and 8.0% of total sequenced *P. multocida* and *M. haemolytica*, respectively). The A2058G mutation in the 23S rRNA gene was detected in two *P. multocida* isolates and linked to resistance against macrolides (Supplementary Table 9). For the remaining sequenced isolates, no relevant 23S rRNA mutation was detected. One *P. multocida* isolate had the A2059G mutation in ~35% of the sequence reads mapped to 23S rRNA. The MICs for tilmicosin and tulathromycin were at 4 μg/ml (both susceptible). No mutations were detected in genes encoding the ribosomal proteins L4 and L22 (*rplD* and *rplV*, respectively). No other macrolide resistance mechanism was evident in the sequenced isolates.

### Effects of Administration of Antimicrobials in the MIC of Respiratory Pathogens

**Figure 2:**
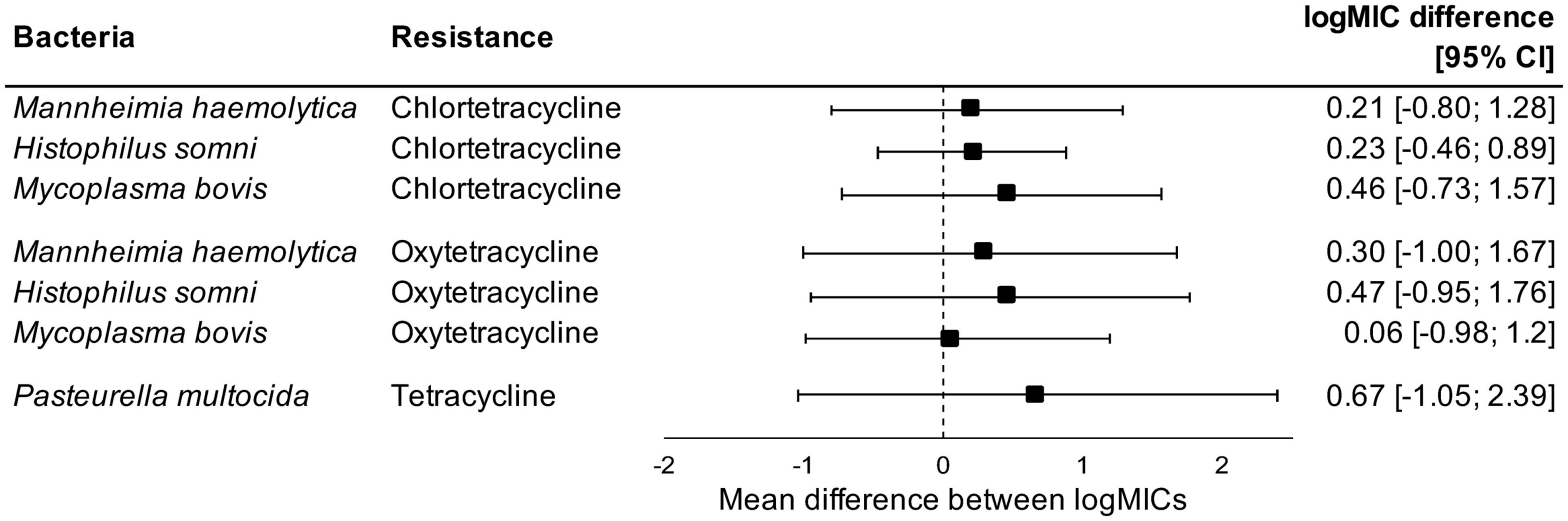
Mean difference between antimicrobial-specific logMICs of bacteria isolated at REPROCESSING from the respiratory tract of calves fed, and not fed tetracyclines at feedlot induction. Model includes observations from all operations, including ones that administered tetracyclines parenterally at induction. Sensitivity analysis indicated no effects of removing observations from operations that administered tetracyclines parenterally at induction.

**Figure 3:**
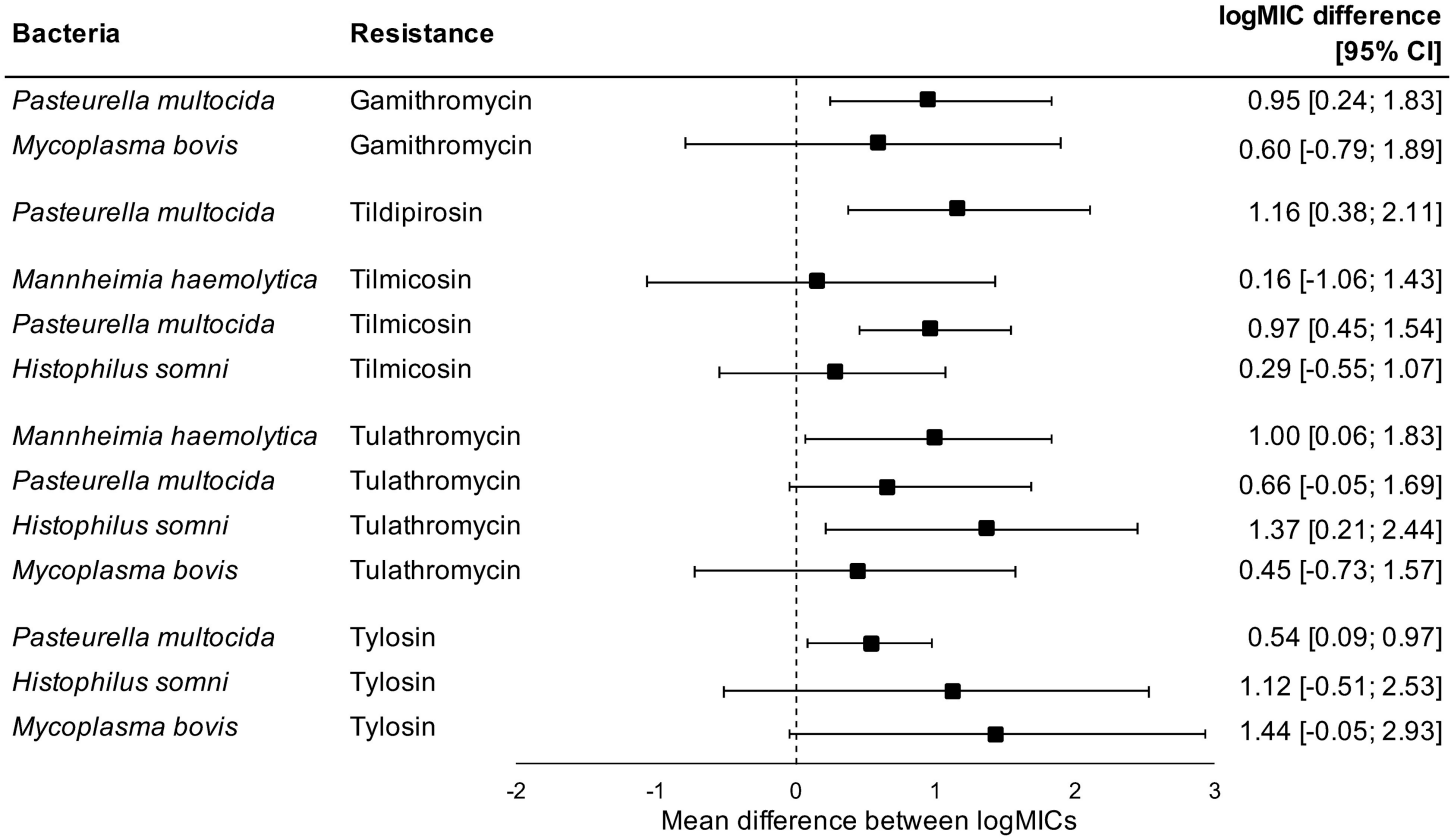
Mean difference between antimicrobial-specific logMICs of bacteria isolated at REPROCESSING from the respiratory tract of animals treated, and not treated parenterally with macrolides at feedlot induction. Model includes observations from all operations, including ones that fed tylosin to control liver abscesses. Sensitivity analysis indicated that effects of parenteral administration of macrolides toward the logMIC of tylosin and gamithromycin in *P. multocida*, and tulathromycin in *H. somni* were statistically significant only when observations from operations that fed tylosin after induction were kept in models.

Administration of tetracyclines at WEANING/INDUCTION was not linked to the MIC of tetracyclines (oxytetracycline, chlortetracycline, and tetracycline) in any respiratory bacteria at REPROCESSING (Figure 2). Effects were independent on the parenteral use of tetracyclines. In contrast, there were clear associations between the use of and reduced susceptibility to macrolides in *P. multocida, M. haemolytica*, and *H. somni* (Figure 3). The parenteral administration of macrolides at WEANING/INDUCTION was linked to an increased MIC of at least one macrolide for each species. In *M. bovis*, the tylosin MIC was on average 1.44 logs higher in bacteria isolated from calves treated parenterally with macrolides at WEANING/INDUCTION vs. the MIC of calves that did not receive macrolides. Yet, no statistically significant association was detected, as the 95% CI ranged from −0.05 to 2.93 (Figure 3). Some of the effects were dependent on inclusion of observations from the two operations that fed tylosin; effects of the parenteral administration of macrolides toward the MIC of tylosin and gamithromycin in *P. multocida* as well as tulathromycin in *H. somni* were detected only when observations from the two operations that fed tylosin after induction were kept in models. The MICs of tilmicosin and tildipirosin in *M. bovis*, and tylosin in *M. haemolytica* were either constant or had a very low variability. Therefore, no modeling was attempted for these antimicrobials.

## Discussion

Here, we report on the prevalence of BRD-associated bacteria in calves from the spring processing to the reprocessing at feedlots. We also determine the AMR profile of respiratory pathogens and explore factors linked to it such as the use and type of antimicrobial used in BRD metaphylaxis. We provide a summary that will integrate the existing literature reporting on AMR of BRD bacteria and explain trends that have been observed in BRD-associated bacteria from feedlot cattle in North America.

There are a number of factors that contribute to clinical BRD in beef cattle, including stressors such as weaning, transportation, and feedlot entry (44), and presence of pathogenic bacteria in the respiratory tract of animals (45). Here, we demonstrate that prevalence of three respiratory pathogens (*P. multocida, M. bovis*, and *M. haemolytica*) increases from spring processing (BRANDING) to reprocessing at feedlots. At least one respiratory pathogen was recovered from >50% of calves at WEANING/INDUCTION and at REPROCESSING, whereas <10% of calves were colonized at BRANDING. *P. multocida* was the most abundant species identified at all three time points, with a marked increase in prevalence at WEANING/INDUCTION that persisted at feedlots. Prevalence of *P. multocida* in clinical BRD appears to be increasing (46). In Canada, *P. multocida* was more prevalent in feedlot cattle with BRD than in healthy control animals (47). Interestingly, prevalence of *P. multocida* at WEANING/INDUCTION was independent of comingling status, which means that an increased prevalence of *P. multocida* was detected for calves sampled early at feedlots as well as for those still at the cow–calf environment (e.g., at weaning). Based on our findings, we hypothesize that animals become increasingly colonized by *P. multocida* prior to or immediately after feedlot placement rather than around 40 days after placement as previously suggested (48). In contrast, *M. bovis* had the highest relative increase in prevalence from WEANING/INDUCTION to REPROCESSING at the feedlot. At BRANDING, prevalence of *M. bovis* was low, in agreement with previous North American studies where the prevalence ranged from 0 to 7% (49, 50). The increased relative abundance of *Mycoplasma* spp. after feedlot placement has been previously described (51), as well as their role in BRD development (45). The highly contagious nature of *M. bovis* among cattle and increased animal density in feedlots have been implicated as the most likely reasons for sharp increases in prevalence of *M. bovis* at feedlots (52).

Comingling of cattle from multiple sources can increase the incidence of respiratory disease (53). We observed some variability of the time of sampling at WEANING/INDUCTION between operations, which reflected producer-specific practices relative to the feedlot induction. This variability was explored in our models by assessing comingling effects. Step et al. (54) reported on associations between commingling and animal health indicators such as antibodies titers to *M. haemolytica* and *P. multocida* and incidence of clinical BRD during a 42-day receiving period. Comingled calves had increased BRD morbidity and were treated earlier and more often with antimicrobials than non-comingled calves, which suggests that comingling can be a risk factor for development of AMR among BRD bacteria. In our study, comingling, results from previous sampling, and co-location of cow–calf operations and feedlots were not associated with prevalence of respiratory pathogens at WEANING/INDUCTION. Additionally, bacteria from comingled and non-comingled cattle had comparable AMR rates at WEANING/INDUCTION. As comingled calves were sampled relatively close to the arrival at feedlots (mean DOF = 9 days, range 2–21 days), we believe that comingling effects in AMR, if present, would manifest later at feeding. Indeed, Step et al. (54) estimated an average of 10.6 days after placement for the first antimicrobial treatment of comingled cattle due to clinical BRD. In contrast, commingling for short periods of time did not significantly impact the nasopharyngeal or tracheal bacterial communities of recently weaned beef calves (51).

*M. haemolytica, P. multocida*, and *H. somni* isolates were susceptible to most antimicrobials tested for which standardized clinical breakpoints were available. None or very few isolates were resistant to florfenicol, fluoroquinolones, and ceftiofur, in agreement to previous findings involving clinically healthy feedlot cattle in Canada (47). Resistance to aminoglycosides was reported in bacteria causing BRD in feedlot cattle in Alberta (55). As inferred from WGS data, ARG profiles of resistant isolates indicated the co-existence of tetracycline [*tet(H)*], neomycin [*aph(3*′*)-Ia*], streptomycin [*aph(6)-Id* and *aph(3*″*)-Ib*], and sulfadimethoxine (*sul2*) determinants in multidrug resistant (MDR) *M. haemolytica* and *P. multocida*. Presence of multiple ARGs in the same isolates suggests existence of integrative and conjugative elements (ICE), which have been previously detected in BRD bacteria (56–58). We confirmed that ICE-associated recombination and conjugation genes were present in MDR isolates. It is unclear what exactly is driving presence of ICE-harboring MDR bacteria in the nasopharynx of beef cattle. In our study, ICE-positive MDR bacteria were isolated from 8 to 17% of sequenced isolates obtained at WEANING/INDUCTION and REPROCESSING, respectively, which suggests that antimicrobial use at induction could be driver of their presence. Nevertheless, it is intriguing that 2 out of 10 sequenced isolates obtained at BRANDING also harbored ICE and were MDR.

We detected the A2058G mutation in the 23S rRNA of two macrolide-resistant *P. multocida*. Macrolide resistance due to rRNA mutations is well-documented in bacteria with a single or multiple rrn operons (59). Bacteria from the *Pasteurellaceae* family associated with BRD typically contain five to six copies of the rrn operon. As more copies harbor the A2058G mutation, a greater increase in the MIC against macrolides is expected. Indeed, a single mutated operon confers only a slight selective advantage in the presence of macrolides, which can be followed by homologous recombination between rrn operons amplifying the proportion of mutant ribosomes, with a corresponding larger increase in macrolide resistance (60). An in-depth analysis of the sequence read data mapped to the wild-type 23S rRNA from *P. multocida* revealed that ~80% of reads from the two macrolide-resistant isolates contained the A2058G SNP, whereas the remaining 20% had the wild-type sequence at the locus, which suggests that four out of five copies of the rrn operons encoded the mutation. Accordingly, the two isolates displayed a high MIC (64 μg/ml) for tilmicosin and tulathromycin.

In *M. bovis*, there was an increased rate of resistance against florfenicol at REPROCESSING in comparison to isolates obtained at WEANING/INDUCTION. Interestingly, florfenicol was the most frequently used antimicrobial after feedlot induction, with four operations reporting at least one treatment with florfenicol. Yet, florfenicol-resistant *M. bovis* were detected in eight feedlots, from which only one reported use of florfenicol. There are two likely explanations to our findings. First, the increase in prevalence of florfenicol-resistant *M. bovis* at REPROCESSING might not be linked to the use of florfenicol at feedlots. Florfenicol-resistant strains could harbor mutations linked to resistance to other antimicrobial classes that were in use at feedlots. Indeed, florfenicol and macrolides target the same ribosomal subunit, and changes in the MIC of tylosin and tilmicosin were noted in florfenicol-resistant strains (61). Unfortunately, as we did not sequence *M. bovis* isolates, we could not infer whether mutations were shared between florfenicol and macrolide-resistant strains. Alternatively, we might have failed to detect antimicrobial therapies with florfenicol at feedlots. From 2008 to 2012, use of phenicols has nearly doubled in Western Canada feedlots, with an estimated use of approximately 1 daily dose for every 10 feedlot cattle in 2012 (62). Additionally, phenicols were among the most frequently used antimicrobials to treat cattle that succumbed to BRD at western Canadian feedlots, where nearly 80% of diseased animals were treated with phenicols (63). Conversely, we observed eight treatments with florfenicol during feeding in nearly 400 feedlot-placed calves. It is conceivable that the use of florfenicol was higher than reported by feedlots, and responsible for increased florfenicol resistance rates in *M. bovis* isolated at REPROCESSING. Our study was done before increased veterinary oversight of antimicrobial use in Canada, as well as prior to the launch of a Canadian fed-cattle antimicrobial surveillance program (64). Under new regulations, medically important antimicrobials are sold under prescription, which facilitates the gathering and recording of antimicrobial usage data, providing a much more reliable metric of antimicrobial exposure for future studies.

High levels of resistance to macrolides in *M. bovis* were detected in our study, in agreement to previous reports (30, 65). A western Canadian study reporting on resistance rates of *M. bovis* recovered from feedlot cattle also demonstrated elevated macrolide-resistance rates, with resistance to various macrolides detected in over 95% of isolates (55). These results suggest that resistance against macrolides in *M. bovis* isolated from the respiratory tract of feedlot cattle in western Canada is widespread. A word of caution is necessary, as currently the CLSI has no approved breakpoints for *M. bovis* isolated from the respiratory tract of beef cattle. Breakpoints adopted herein were defined based on a combination of historical MIC data, previous reports and current breakpoints for other respiratory pathogens of cattle (30). When MIC_50_ of macrolides were inspected, our estimates were comparable to ones reported from France and Japan in 2008–2012 (11, 66), and higher than values reported from the US in 2002–2003 (29).

Susceptibility of *M. bovis* from feedlot cattle to macrolides has decreased over the last decades in France (11). In Canada, although we currently lack definitive data to support a sustained increase in AMR rates of *M. bovis*, there is some evidence suggesting an increase in resistance against tulathromycin over the last decade (30). Additionally, a study done in 2007 and 2008 involving *M. bovis* from dead and sick cattle from a single feedlot in western Canada reported lower MIC_50_ values of tulathromycin than those observed in our study (67), which also suggests that macrolide resistance of *M. bovis* from western Canadian feedlots is on the rise. Such trends could be driven by the increased use of macrolides in western Canadian feedlots for metaphylaxis and the prevention of liver abscesses (68). The link between use of macrolides in feedlot cattle and resistance against tulathromycin in *M. bovis* isolated from the respiratory tract of feedlot cattle was recently explored. *M. bovis* isolated from cattle raised without antimicrobials had diminished MIC of tulathromycin in comparison to bacteria from cattle exposed to macrolides (12). In our study, the incidence of tulathromycin-resistant *M. bovis* at REPROCESSING was 5.3 times higher in feedlots that treated calves parenterally with macrolides at induction in comparison to feedlots not using macrolides (*p* = 0.04 in operation-conditional Poisson models; results not shown).

We detected significant associations between parenteral use of and increased MICs against macrolides in *P. multocida, H. somni*, and *M. haemolytica* at REPROCESSING. We were not able to infer whether effects were also evident for the in-feed administration of macrolides; the two operations that fed tylosin after induction as prevention of liver abscesses also inducted with macrolides. Nonetheless, by excluding observations from operations in which tylosin was administered to calves *via* feed, some of the associations previously detected were lost, which suggests that the in-feed administration of tylosin will also play a role in the selection of respiratory bacteria with increased MIC against macrolides. Macrolides are antimicrobials of critical importance to human health according to the WHO (9). It is unknown if and to what extent the presence of respiratory bacteria with increased resistance to macrolides in beef cattle represents a threat to humans or to the environment. Furthermore, we are not able to estimate the likely impacts of increased MICs of macrolides on the therapeutic effectiveness of antimicrobials against BRD, especially if increases incur under breakpoints used to define clinical resistance. Nevertheless, our findings are extremely valuable to interpret trends of susceptibility in BRD bacteria from North American feedlots. Decreases in susceptibility to macrolides in *P. multocida, H. somni*, and *M. haemolytica* have been documented (10). Based on our findings, we hypothesize that the use of macrolides at feedlots is impacting the susceptibility of respiratory bacteria against macrolides.

Conversely, the in-feed administration of tetracyclines as metaphylaxis was not associated with antimicrobial-specific MICs in respiratory bacteria isolated from feedlot cattle at REPROCESSING. Levels of AMR are expected to increase after antimicrobial use, but this pattern was not evident when chlortetracycline was fed in our study. The lack of an association could be due to an already widespread prevalence of resistance to tetracyclines in respiratory bacteria of feedlot cattle in western Canadian feedlots. Timsit et al. (47) studied the prevalence of AMR in *P. multocida, H. somni*, and *M. haemolytica* recovered from the respiratory tract of healthy and sick cattle in Canadian feedlots. Resistance to oxytetracycline was widespread, regardless of animal category or bacteria species. Yet, most of our isolates were susceptible to tetracyclines, and resistance levels were much lower than reported previously in western Canada (55). Alternatively, we could have missed rapid, transient increases of AMR following use of chlortetracycline in feed. Indeed, cattle exposed to a 5-day therapy with chlortetracycline in feed had increased levels of tetracycline-resistant *Escherichia coli* in feces 5 days post-treatment in comparison to unexposed animals, with no differences between groups detected >27 days post-treatment (69). Our median DOF at REPROCESSING was 139 days, which means that transient increases of AMR in respiratory bacteria, if any, were probably not captured by our study.

Our findings suggest that, under One Health lens, tetracyclines fed at induction will have a minimal long-term impact in prevalence of AMR in respiratory bacteria, which could support the sustained use of tetracyclines in feedlots as BRD metaphylaxis. From an animal health perspective, two meta-analysis explored the effects of metaphylaxis on BRD in beef cattle. Abell et al. (15) identified differences between classes of antimicrobials used as metaphylaxis, with macrolides outperforming tetracyclines, regardless of the duration of the feeding period. In contrast, Baptiste and Kyvsgaard (70) did not detect differences in efficacy of these antimicrobial classes against BRD; macrolides were superior to tetracyclines only when used as a prophylaxis (medication of asymptomatic cattle upon arrival at feedlot), but not as metaphylaxis. Altogether, further consideration should be given to the in feed use of tetracyclines as metaphylaxis, including the identification of specific circumstances in which these antimicrobials should be recommended as first choice in prevention of BRD.

As the use of macrolides at feedlot induction was linked to the MIC of bacteria isolates at REPROCESSING, we were surprised by the lack of differences in prevalence of macrolide resistance between sampling points. This contradiction is a consequence of at least four factors. First, not all feedlots administered macrolides at induction, and resistance to macrolides could have been dependent on the use of macrolides. Indeed, the incidence of tulathromycin-resistant *M. bovis* was statistically higher at REPROCESSING in comparison to WEANING/INDUCTION only in feedlots that induced with macrolides (1.4 resistant isolates/100 calves at WEANING/INDUCTION vs. 5.2 resistant isolates/100 calves at REPROCESSING in feedlots that used macrolides at induction, *p* < 0.05 in operation-conditional Poisson models; results not shown). Second, increases in MIC could have occurred under clinical breakpoints used to define macrolide resistance, particularly for species with low resistance levels (*M. haemolytica, H. somni*, and *P. multocida*). Surveillance data have demonstrated substantial year-to-year variation in the MIC of some macrolides, but with values generally below the specific criteria for defining resistance (71). The clinical implications of sub-breakpoints MIC increases remain to be demonstrated, but such increases could partially account for historical trends observed in MIC data. Third, we could have lacked power to detect specific differences in AMR rates between sampling points. Despite lack of statistical significance, all macrolides resistance rates in *P. multocida* isolated at REPROCESSING were higher in comparison to resistance rates at other sampling points. Finally, Bayesian models for MIC data can be used even in absence of clinical or epidemiological susceptibility breakpoints. For antimicrobials in which breakpoints have not been defined (e.g., tylosin in *P. multocida, H. somni*, and *M. haemolytica*), we were not able to compare resistance rates between sampling points, which could contrast with findings obtained from Bayesian models.

We provided important results from a longitudinal study that can be used to inform strategies to optimize use of antimicrobials in beef cattle. Nevertheless, our findings should be considered in the face of study limitations and caveats. In a cohort study, loss to follow-up can negatively affect the internal validity of the study if the probability of missingness is dependent on the observed values, values that would have been observed, or both. Producers were selected, in part, by their usual management strategy of retaining ownership of their cattle through to slaughter. However, more cattle than expected were sold before the end of the study, or producers divided study calves among a number of pastures or pens and animals were not available for sampling. Such challenges are not uncommon when working with commercial operations. Regardless, we believe our findings are internally valid for at least three reasons. First, decisions to sell cattle or divide the study calves among several pastures were based on economics and management terms and not on animal health. There is limited evidence to suggest that the probability of not being sampled was dependent on presence of respiratory bacteria that were either observed or that would have been observed. Our missing data were most likely generated at random, also given results from the comparison between some of the completers and non-completers (discussed in the Statistical Analysis). Second, we used operation-conditional models, meaning that estimates and comparisons reported were conditional on the operation. These models are efficient at dealing with missing data that are generated randomly (e.g., missing completely at random or missing at random). Hence, any impact arising from imbalances in the number of samples collected per operation was minimized, assuming that, within each operation, the probability of not being sampled was independent of observed values. Third, for prevalence estimates, we used multiple imputation models that included results from previous sampling point(s) and operation random effects. This approach is very efficient to increase the study power and therefore minimize impacts of missing data. A second limitation arises from the use of sensitivity and specificity estimates in our prevalence models. It is unclear whether the sensitivity and specificity values of DNPS were adequate for detection of *H. somni* and *P. multocida* in the respiratory tract of calves. Unfortunately, to our knowledge there is no study reporting on the characteristics of DNPS to detect the two species in the respiratory tract of beef cattle.

In summary, *P. multocida* was the most prevalent bacteria in the respiratory tract of beef calves from spring processing to reprocessing at feedlots, followed by *M. haemolytica, M. bovis*, and *H. somni*. For *M. bovis*, a sharp increase in prevalence was detected at REPROCESSING, whereas for *P. multocida*, an increase in prevalence occurred at WEANING/INDUCTION and persisted during feeding. Comingling was not associated with prevalence of any respiratory pathogen at feedlot induction. Resistance levels were generally low, with a few exceptions. In *M. bovis*, resistance against macrolides was prevalent, with the majority of isolates resistant against tildipirosin, tilmicosin, and tylosin. There was limited evidence to support an increase in resistance rates from the spring processing to reprocessing at feedlots in *M. haemolytica, P. multocida*, and *H. somni*, although macrolide resistance rates were consistently higher in *P. multocida* at REPROCESSING. In *M. bovis*, increased florfenicol resistance rates were detected at REPROCESSING. Metaphylactic administration of tetracyclines at feedlot induction was not linked to the MIC of tetracyclines in any respiratory bacteria at REPROCESSING. Conversely, there were clear associations between the parenteral use of macrolides as metaphylaxis, and increased MIC in *P. multocida, M. haemolytica*, and *H. somni*. We hypothesize that the use of macrolides such as tulathromycin at feedlot induction is responsible for historical changes in macrolides MIC data of respiratory bacteria isolated from post-arrival cattle in feedlots.

## Data Availability Statement

The datasets presented in this study can be found in online repositories. The sequencing data of isolates used in this study have been submitted to the NCBI (BioProject ID: PRJNA720670/PRJNA313047).

## Ethics Statement

The animal study was reviewed and approved by Lethbridge Research and Development Centre's Animal Care Committee. Written informed consent for participation was not obtained from the owners because consent was provided verbally as a result of communication between veterinary practitioners and their clients.

## Author Contributions

RA, TM, EH, and CD conceived and designed the study. CD, RA, SA-L, and EH were involved in sample and data collection. RZ worked on the sequencing data. DN performed statistical analysis and worked in the interpretation of results, and wrote the first draft of the manuscript. RA, TM, EH, SA-L, RZ, and CD revised the document for important intellectual content. All authors gave approval of the final version to be published and agreed to be accountable for all aspects of the work.

## Funding

This study received funding from Alberta Beef Producers (ABP) and Alberta Livestock and Meat Agency Ltd. (ALMA). The funder was not involved in the study design, collection, analysis, interpretation of data, the writing of this article or the decision to submit it for publication.

## Conflict of Interest

CD and EH are part owner and managing partners of Veterinary Agri-Health Services. Veterinary Agri-Health Services is a health management oriented veterinary practice based in Alberta that provides professional services to feedlot and cow/calf operations across western Canada. The remaining authors declare that the research was conducted in the absence of any commercial or financial relationships that could be construed as a potential conflict of interest.

## Publisher's Note

All claims expressed in this article are solely those of the authors and do not necessarily represent those of their affiliated organizations, or those of the publisher, the editors and the reviewers. Any product that may be evaluated in this article, or claim that may be made by its manufacturer, is not guaranteed or endorsed by the publisher.
