# Supplementary material for: Prevalence, Risk Factors, and Antimicrobial Resistance Profile of Respiratory Pathogens Isolated From Suckling Beef Calves to Reprocessing at the Feedlot: A Longitudinal Study
Source: Front Vet Sci. 2021 Nov 2;8:764701. doi: 10.3389/fvets.2021.764701 (PMC8596561; doi:10.3389/fvets.2021.764701)
Supplement: Supplementary file 1 [file Data_Sheet_1.PDF]

frontiers

Supplementary Material

1 Supplementary Methods

Deep Nasopharyngeal Swab Protocol

1. Restrain the animal's head.
2. Wipe the external nares with single use towel (paper or cloth is acceptable).
3. Estimate the distance from the external nares and medial canthus of the eye – this is the distance to insert the guarded swab.
4. Direct the guarded swab medially and ventrally as it is inserted into the nasal cavity. Once the estimated insertion point is reached, fully extend the swab through the guarded tip.
5. To sample, use several short back-and-forth / circular motions of the swab.
6. Only one nostril (right or left) should be sampled.
7. After collecting the sample, withdrawal the swab into the guard tube, then remove entire apparatus from the nasal cavity.
8. Place the swab into a Cary Blair transport tube by breaking at the scored area of the swab.
9. Label the transport tube with animal identification and collection date.
10. Place transport tubes on cold packs immediately following collection.
11. All samples should be refrigerated and delivered by courier to the Lethbridge Research Centre weekly.

2 Supplementary Figures and Tables

2.1 Supplementary Figures

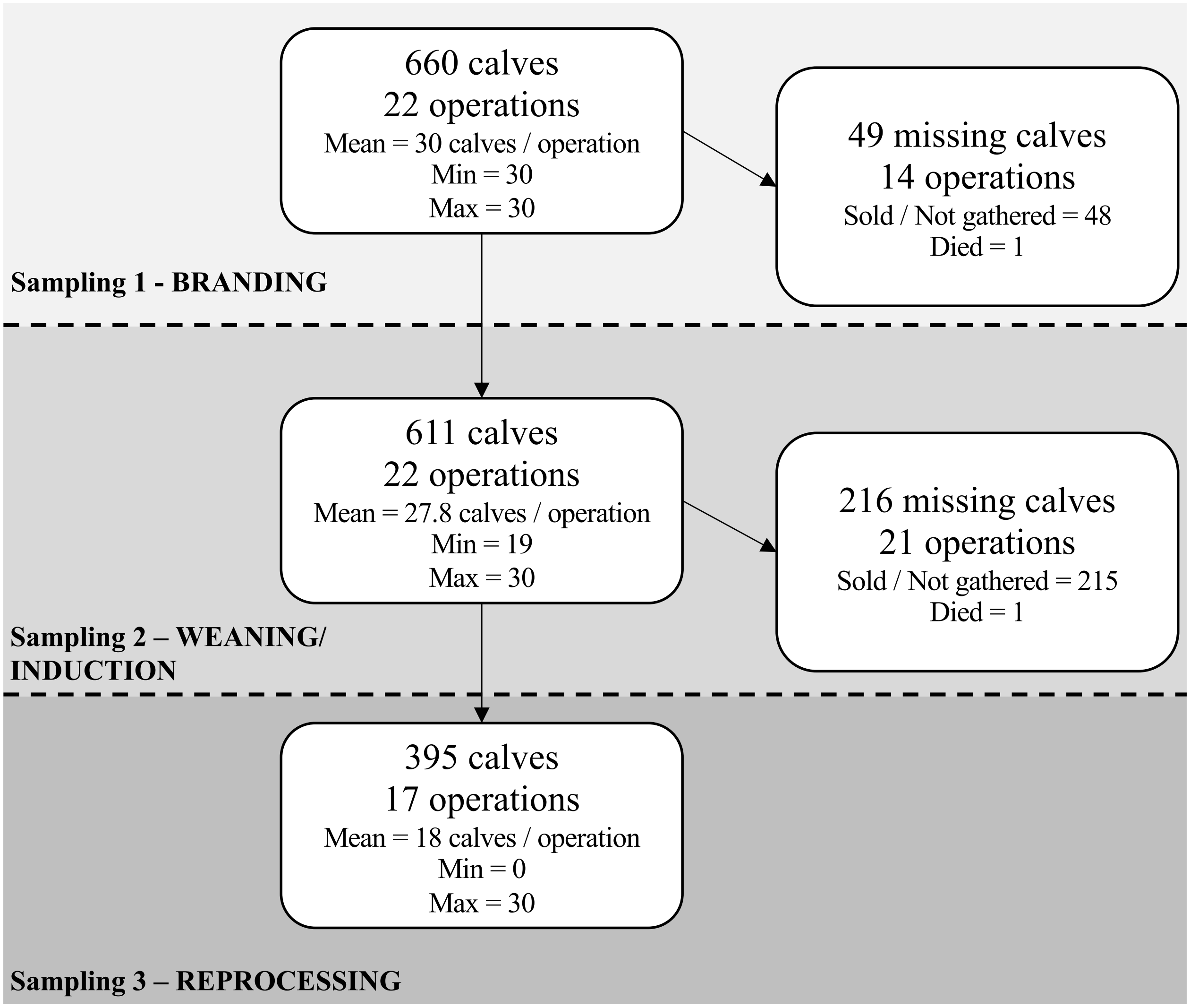

Supplementary Figure 1. Summary of animals and operations included at each sampling.

2.2 Supplementary Tables

Supplementary Table 1. Antimicrobials, range of concentrations tested, and breakpoints used in *Mannheimia haemolytica, Pasteurella multocida*, and *Histophilus somni* isolated from the respiratory tract of beef cattle

**Class**	**Antimicrobial**	**Test range (µg/mL)[1]**	**Breakpoints (µg/mL)[2]**		
			**S**	**I**	**R**
Aminocyclitol	Spectinomycin	8 – 64	≤ 32	64	≥ 128
Aminoglycoside	Gentamycin	1 – 16	-	-	-
	Neomycin	4 – 32	-	-	-
Cephalosporin	Ceftiofur	0.25 – 8	≤ 2	4	≥ 8
Sulfonamide / folate pathway inhibitor	Trimethoprim/ sulfamethoxazole	2/38	-	-	-
Fluoroquinolone	Danofloxacin	0.12 – 1	≤ 0.25[3]	0.5[3]	≥ 1[3]
	Enrofloxacin	0.12 – 2	≤ 0.25	0.5 – 1	≥ 2
Lincosamide	Clindamycin	0.25 – 16	-	-	-
Macrolide	Tilmicosin	4 – 64[4]	≤ 8[5]	16[5]	≥ 32[5]
	Tulathromycin	1 – 64[6]	≤ 16	32	≥ 64
	Tylosin tartrate	0.5 – 32	-	-	-
	Gamithromycin[7]	1 – 8	≤ 4	8	≥ 16
	Tildipirosin[7]	1 – 16	≤ 8	16	≥ 32
Phenicol	Florfenicol	0.25 – 8	≤ 2	4	≥ 8
Pleuromutilin	Tiamulin	0.5 – 32	-	-	-
Sulfonamide	Sulfadimethoxine	256	-	-	-
Tetracycline	Chlortetracycline[8]	0.5 – 8	-	-	-
	Oxytetracycline[8]	0.5 – 8	≤ 2[9]	4[9]	≥ 8[9]
	Tetracycline[7]	0.5 – 8	≤ 2	4	≥ 8
Penicillin	Ampicillin	0.25 – 16	≤ 0.03[10]	0.06 – 0.12[10]	≥ 0.25[10]
	Penicillin	0.12 – 8	≤ 0.25	0.5	≥ 1

[1]For *H. somni*, concentrations tested were half of values listed;
[2]Cattle-specific breakpoints defined according to the VET01S document from the Clinical and Laboratory Standards Institute, 5th edition (1). S = susceptible; I = intermediate; R = resistant. A dash (-) denotes absence of defined clinical breakpoints for that antimicrobial in all three bacteria species, unless otherwise specified;
[3]Breakpoint developed for *M. haemolytica* and *P. multocida* was used for *H. somni* (2);
[4]For *P. multocida*, test range was 2-16 µg/mL;
[5]Breakpoint developed for *M. haemolytica* was used for *P. multocida* and *H. somni* (2);
[6]For *P. multocida*, test range was 8-64 µg/mL;
[7]Tested in *P. multocida*, exclusively;
[8]Tested in *M. haemolytica* and *H. somni*, exclusively;
[9]Breakpoints assumed to be identical to the tetracycline ones;
[10]Breakpoints not used given that concentrations tested were insufficient to distinguish between susceptible and non-susceptible bacteria.

Supplementary Table 2. Antimicrobials, range of concentrations tested, and breakpoints used in *Mycoplasma bovis* isolated from the respiratory tract of beef cattle

Class	Antimicrobial	Test range (µg/mL)	Breakpoints (µg/mL)[1]		
			S	I	S
Fluoroquinolone	Enrofloxacin	0.12 – 128	≤ 0.25	0.5 – 1	≥ 2
Macrolide	Gamithromycin	0.25 – 256	≤ 4	8	≥ 16
	Tildipirosin	0.12 –128	≤ 8	-	≥ 16
	Tilmicosin	0.5 – 256	≤ 8	16	≥ 32
	Tulathromycin	0.25 – 256	≤ 16	32	≥ 64
	Tylosin tartrate	0.5 – 128	≤ 4	-	≥ 8
Phenicol	Florfenicol	0.25 – 256	≤ 2	4	≥ 8
Tetracycline	Chlortetracycline	0.5 – 256	≤ 4	-	≥ 8
	Oxytetracycline	0.5 – 256	≤ 4	-	≥ 8

[1]Cattle-specific breakpoints defined according to Jelinski, Kinnear (3). S = susceptible; I = intermediate; R = resistant;
A dash (-) denotes absence of an intermediate category for that antimicrobial.

Supplementary Table 3. Characteristics of cow-calf operations enrolled and samples collected

Cow-calf operation number	Feedlot on site	Number of samples at branding	Number of samples at weaning/ feedlot induction	Comingled sample at weaning/ feedlot induction	Number of samples at reprocessing	Comingled sample at reprocessing
1	Yes	30	29	No	27	Yes
2	Yes	30	29	Yes	29	Yes
3	Yes	30	30	No	14	Yes
4	No	30	19	No	0	Yes
5	No	30	28	No	26	Yes
6	No	30	28	No	0	Yes
7	Yes	30	28	Yes	25	Yes
8	Yes	30	30	No	0	Yes
9	No	30	25	No	25	No
10	Yes	30	26	No/Yes[1]	0	Yes
11	Yes	30	30	Yes	22	Yes
12	Yes	30	28	No/Yes[1]	15	Yes
13	Yes	30	30	Yes	7	Yes
14	Yes	30	23	No	0	Yes
15	Yes	30	26	Yes	25	Yes
16	Yes	30	30	No	30	Yes
17	Yes	30	30	No	16	Yes
18	Yes	30	29	No	23	Yes
19	No	30	24	Yes	29	Yes
20	No	30	30	Yes	29	Yes
21	Yes	30	30	No	25	No
22	Yes	30	29	No	28	Yes

[1]Animal groups processed on different days

Supplementary Table 4. Mean, lower and upper limits of prior distributions used to account for potential misclassification of disease-positive animals (sensitivity) according to the pathogen

**Pathogen**	**Distribution**	**Mean**	**Limit**		**Reference**
			**Lower**	**Upper**	
M. haemolytica[1]	β (22.69, 2.29)	0.944	0.894	1	(4)
P. multocida[2]	β (22.69, 2.29)	0.944	0.894	1	
H. somni[2]	β (22.69, 2.29)	0.944	0.894	1	
M. bovis[3]	β (8.34, 3.09)	0.778	0.728	0.828	

[1]In Godinho, Sarasola (4), 18 calves with BRD symptoms had *M. haemolytica* isolated from at least one of the following: nasopharyngeal swab, lung lavage, lung tissue and lung swab. From this total, 17 calves (94.4%) had the bacteria detected in deep nasopharyngeal swabs;
[2]Sensitivity assumed to be identical to the *M. haemolytica* one;
[3]In Godinho, Sarasola (4), 18 calves with BRD symptoms had *M. bovis* isolated from at least one of the following: nasopharyngeal swab, lung lavage, lung tissue and lung swab. From this total, 14 calves (77.8%) had the bacteria detected in deep nasopharyngeal swabs.

Supplementary Table 5. MIC 50 and MIC 90 (µg/mL) of *Mannheimia haemolytica* isolates collected at BRANDING (*n* = 1), WEANING/INDUCTION (*n* = 30), and REPROCESSING (*n* = 27)

**Antimicrobial**	**BRANDING**		**WEANING/INDUCTION**		**REPROCESSING**	
	**MIC 50**	**MIC 90**	**MIC50**	**MIC 90**	**MIC 50**	**MIC 90**
Spectinomycin	32	32	32	64	32	32
Gentamycin	2	2	4	4	2	2
Neomycin	<=4	<=4	8	8	8	8
Ceftiofur	0.5	0.5	0.5	1	<=0.25	<=0.25
Trimethoprim/ sulfa	<=2/38	<=2/38	<=2/38	>2/38	<=2/38	<=2/38
Danofloxacin	<=0.12	<=0.12	<=0.12	<=0.12	<=0.12	<=0.12
Enrofloxacin	<=0.12	<=0.12	<=0.12	<=0.12	<=0.12	<=0.12
Clindamycin	>16	>16	>16	>16	8	>16
Tilmicosin	<=4	<=4	8	16	8	16
Tulathromycin	4	4	4	8	4	16
Tylosin tartrate	>32	>32	>32	>32	>32	>32
Florfenicol	1	1	1	1	1	1
Tiamulin	32	32	32	32	16	32
Sulfadimethoxine	>256	>256	>256	>256	>256	>256
Chlortetracycline	1	1	1	1	<=0.5	1
Oxytetracycline	1	1	1	1	<=0.5	1
Ampicillin	<=0.25	<=0.25	<=0.25	<=0.25	<=0.25	<=0.25
Penicillin	0.25	0.25	0.25	0.25	0.25	0.5

No standard breakpoint   Susceptible   Intermediate

Supplementary Table 6. MIC 50 and MIC90 (µg/mL) of *Histophilus somni* isolates collected at WEANING/INDUCTION (n = 10) and REPROCESSING (n = 20)

**Antimicrobial**	**WEANING/INDUCTION**		**REPROCESSING**	
	**MIC50**	**MIC 90**	**MIC 50**	**MIC 90**
Spectinomycin	16	32	16	32
Gentamycin	2	4	2	2
Neomycin	8	>16	8	>16
Ceftiofur	<=0.125	<=0.125	<=0.125	<=0.125
Trimethoprim/ sulfa	<=1/19	<=1/19	<=1/19	<=1/19
Danofloxacin	<=0.06	0.125	<=0.06	<=0.06
Enrofloxacin	<=0.06	<=0.06	<=0.06	<=0.06
Clindamycin	0.5	2	0.5	1
Tilmicosin	<=2	4	<=2	4
Tulathromycin	2	8	2	8
Tylosin tartrate	2	4	2	4
Florfenicol	<=0.125	0.25	<=0.125	<=0.125
Tiamulin	1	2	0.5	2
Sulfadimethoxine	>128	>128	>128	>128
Chlortetracycline	<=0.25	<=0.25	<=0.25	<=0.25
Oxytetracycline	<=0.25	0.5	<=0.25	0.5
Ampicillin	<=0.125	0.25	<=0.125	0.25
Penicillin	<=0.06	0.125	<=0.06	0.125

No standard breakpoint   Susceptible

Supplementary Table 7. MIC 50 and MIC90 (µg/mL) of *Mycoplasma bovis* isolates collected at BRANDING (n = 2), WEANING/INDUCTION (n = 19) and REPROCESSING (n = 28)

Antimicrobial	BRANDING		WEANING/INDUCTION		REPROCESSING	
	MIC 50	MIC 90	MIC50	MIC 90	MIC 50	MIC 90
Enrofloxacin	<=0.12	<=0.12	<=0.12	<=0.12	<=0.12	<=0.12
Gamithromycin	128	128	64	256	128	256
Tildipirosin	>128	>128	>128	>128	>128	>128
Tilmicosin	256	256	256	256	256	256
Tulathromycin	4	16	16	256	64	256
Tylosin tartrate	16	32	16	>128	32	>128
Florfenicol	2	4	2	4	4	8
Chlortetracycline	2	4	0.5	8	4	8
Oxytetracycline	2	4	1	8	4	8

No standard breakpoint Susceptible Intermediate Resistant

Supplementary Table 8. MIC 50 and MIC90 (μg/mL) of *Pasteurella multocida* isolates collected at BRANDING (n = 24), WEANING/INDUCTION (n = 88), and REPROCESSING (n = 38)

**Antimicrobial**	**BRANDING**		**WEANING/INDUCTION**		**REPROCESSING**	
	**MIC 50**	**MIC 90**	**MIC50**	**MIC 90**	**MIC 50**	**MIC 90**
Spectinomycin	16	32	16	64	32	128
Gentamycin	4	4	4	8	4	8
Neomycin	16	16	16	64	16	64
Ceftiofur	<=0.25	<=0.25	<=0.25	<=0.25	<=0.25	<=0.25
Trimethoprim/ sulfa	<=2/38	<=2/38	<=2/38	<=2/38	<=2/38	<=2/38
Danofloxacin	<=0.12	<=0.12	<=0.12	<=0.12	<=0.12	<=0.12
Enrofloxacin	<=0.12	<=0.12	<=0.12	<=0.12	<=0.12	<=0.12
Clindamycin	>16	>16	>16	>16	>16	>16
Gamithromycin	<=1	<=1	<=1	<=1	<=1	2
Tildipirosin	<=1	<=1	<=1	2	<=1	2
Tilmicosin	4	8	4	8	4	8
Tulathromycin	<=8	<=8	<=8	<=8	<=8	<=8
Tylosin tartrate	32	>32	32	>32	32	>32
Florfenicol	<=0.25	0.5	<=0.25	0.5	0.5	0.5
Tiamulin	32	32	16	32	16	32
Sulfadimethoxine	>256	>256	>256	>256	>256	>256
Tetracycline	<=0.5	1	<=0.5	1	<=0.5	8
Ampicillin	<=0.25	<=0.25	<=0.25	<=0.25	<=0.25	<=0.25
Penicillin	<=0.12	<=0.12	<=0.12	<=0.12	<=0.12	0.25

No standard breakpoint | Susceptible | Intermediate | Resistant

Supplementary Table 9. Proportion of antimicrobial resistant isolates according to the presence or absence of antimicrobial resistance genes

Bacteria	Gene	Resistance	Proportion		*P*
			Gene -	Gene +	
M. haemolytica	*tetH*	Oxytetracycline	0	100	0.004
P. multocida	*aadA31*	Spectinomycin	0	100	< 0.001
	tetH	Tetracycline	0	100	< 0.001
	A2058G	Tildipirosin	0	100	0.003
	A2058G	Gamithromycin	4	100	0.009
	A2058G	Tilmicosin	8	100	0.02
	A2058G	Tulathromycin	0	100	0.003

3 Supplementary References

1. Clinical and Laboratory Standards Institute. *Performance Standards for Antimicrobial Disk and Dilution Susceptibility Tests for Bacteria Isolated From Animals; Approved Standard* 4th ed. VET01-A4. Institute CaLS, editor. Wayne, PA, USA (2013)
2. Clinical and Laboratory Standards Institute. *Understanding Susceptibility Test Data as a Component of Antimicrobial Stewardship in Veterinary Settings* 1st ed. Institute CaLS, editor. Wayne, PA (2019)
3. Jelinski M, Kinnear A, Gesy K, Andres-Lasheras S, Zaheer R, Weese S, et al. Antimicrobial sensitivity testing of *Mycoplasma bovis* isolates derived from western Canadian feedlot cattle. *Microorg* (2020) 8. doi:10.3390/microorganisms8010124
4. Godinho KS, Sarasola P, Renoult E, Tilt N, Keane S, Windsor GD, et al. Use of deep nasopharyngeal swabs as a predictive diagnostic method for natural respiratory infections in calves. *Vet Rec* (2007) 160:22-5. doi:10.1136/vr.160.1.22
